# Xenobiology for the Biocontainment of Synthetic Organisms: Opportunities and Challenges

**DOI:** 10.3390/life14080996

**Published:** 2024-08-10

**Authors:** Lucía Gómez-Tatay, José Miguel Hernández-Andreu

**Affiliations:** 1 Institute of Life Sciences, Universidad Católica de Valencia San Vicente Mártir, 46001 Valencia, Spain; lucia.gomez@ucv.es; 2 Grupo de Investigación en Medicina Molecular y Mitocondrial, Universidad Católica de Valencia San Vicente Mártir, 46001 Valencia, Spain

**Keywords:** biocontainment, xenobiology, orthogonal, unnatural, synthetic biology, genetic firewalls, biosafety

## Abstract

Since the development of recombinant DNA technologies, the need to establish biosafety and biosecurity measures to control genetically modified organisms has been clear. Auxotrophies, or conditional suicide switches, have been used as firewalls to avoid horizontal or vertical gene transfer, but their efficacy has important limitations. The use of xenobiological systems has been proposed as the ultimate biosafety tool to circumvent biosafety problems in genetically modified organisms. Xenobiology is a subfield of Synthetic Biology that aims to construct orthogonal biological systems based on alternative biochemistries. Establishing true orthogonality in cell-based or cell-free systems promises to improve and assure that we can progress in synthetic biology safely. Although a wide array of strategies for orthogonal genetic systems have been tested, the construction of a host harboring fully orthogonal genetic system, with all parts operating in an orchestrated, integrated, and controlled manner, still poses an extraordinary challenge for researchers. In this study, we have performed a thorough review of the current literature to present the main advances in the use of xenobiology as a strategy for biocontainment, expanding on the opportunities and challenges of this field of research.

## 1. Background

According to Wooster, the term “xenobiology” was first used by Robert Heinlein around 1954 “in designating non-terrestrial things, concepts, and fields of study” [1]. Today, xenobiology is considered a subfield of Synthetic Biology, a scientific discipline that applies engineering principles to biology. Xenobiology specifically aims to construct orthogonal biological systems, such as bacteria relying on an extended genetic code. Other subfields of Synthetic Biology work, for example, include obtaining standard biological parts to be used in the building of biological systems, developing new functions in microorganisms, minimizing the genome of natural bacteria, synthesizing whole genomes, or manufacturing whole cells (protocells) [2,3]. Xenobiology is then a very particular subfield within Synthetic Biology, since it is based in developing an alternative biochemistry of life, different from that which operates naturally in organisms. According to the Scientific Committees of the European Commission, the Scientific Committee on Consumer Safety (SCCS), the Scientific Committee on Health and Environmental Risks (SCHER), and the Scientific Committee on Emerging and Newly Identified Health Risks (SCENIHR), xenobiology can be defined as “the design, engineering and production of biological systems with non-natural biochemistry and/or alternative genetic codes” and tries to understand the origin of life (why life has evolved the way it has and not differently), to produce economically interesting organisms or compounds with useful functions and to implement new types of biocontainment, impeding horizontal gene flow between natural and engineered organisms [4].

In a review on xenobiology by Schmidt and Pei, it is claimed that xenobiology has the main goal of responding to fundamental questions on the chemistry of life: can biological systems function with an alternative genetic code composed of XNA, or with proteins containing ncAAs, or even with both components? That is to say that xenobiological research has a tight relationship with studies on the origin of life, including the development of the genetic code [5]. The study of the causes and the processes that have determined the development of the actual genetic code is fundamental when thinking about expanding it. The “Alanine World” theory explains that protein biosynthesis started with a reduced set of amino acids restricted to codons containing GC: glycine, proline, alanine, and one cationic species (now: arginine). Interestingly, subsequent expansion of the genetic code was exclusively based on structural derivatives of alanine instead of relying on all four amino acids equally. This was due to the α-helical propensity of alanine. In this way, the protein world we know can be called “the Alanine World” [6]. The question now is whether we can artificially direct parallel protein worlds since the other starting amino acids can also be a feasible starting point for protein biosynthesis. Xenobiology will help us answer this question. According to Kubyshkin and Budisa, escaping the Alanine World is a complex and long-term task. However, once realized, this will allow us to answer many fundamental questions about the origins of life, while also delivering completely distinct and biotechnological innovations [7].

Since the development of recombinant DNA technologies in the 1970s, the need for biosafety and biosecurity measures to control the genetically modified organisms (GMOs) has been evident, as it was first outlined in the 1975 Asilomar conference on recombinant DNA [8]. Among these measures, developing effective biological barriers that limit the survival of GMOs in the environment (biocontainment measures) remains challenging [9]. There are options such as auxotrophies, or conditional suicide switches, but these can be affected by metabolic cross-feeding or genetic mutation, respectively. Xenobiology could provide an interesting approach to circumvent these difficulties, for example generating synthetic auxotrophs whose growth depends on exogenously supplied synthetic amino acids (sAAs) [10]. While most of the Synthetic Biology subfields try to fine-tune the newly constructed pathways while still relying on the same biochemistry naturally occurring in the organism, xenobiology, on the other hand, tries to achieve this “orthogonality” by changing the biochemistry of synthetic life (both genetics and metabolism), so that the synthetic systems cannot interfere with naturally occurring ones [11]. This separation can be achieved by means of xenonucleic acids, which are nucleic acids different from both DNA and RNA, for example, nucleic acids composed of unnatural bases, or building xenoproteins using noncanonical amino acids [12]. It has even been proposed, to overcome the central dogma of biology, to design some other type of alternative paradigm that does not follow the DNA-RNA-protein pathway [2]. Depending on the modifications performed, these organisms can be classified as CMOs, GROs, or CMGROs. CMOs are Chemically Modified Organisms, which have altered biochemical building blocks (e.g., nucleic acids, amino acids). GROs are Genomically Recoded Organisms, with a modified genetic code. CMGROs are Chemically Modified and Genomically Recoded Organisms, with both alternative building blocks and alternative codes [13]. These organisms are expected to “constitute an informational enclave since horizontal gene transfer with natural cells is (at least partially) impaired” [13]. Therefore, xenobiology could be the ultimate biosafety tool, as far as the use of synthetic information-storing biopolymers will ensure the “invisibility” of orthogonal systems to the natural counterparts [14].

This study reviews and discusses the principal approaches up to date in biocontainment of xenobiology and elaborates on the opportunities that this new field of research offers, as well as the main challenges that this area poses.

## 2. Opportunities of Xenobiology in Biocontainment

**Figure 1:**
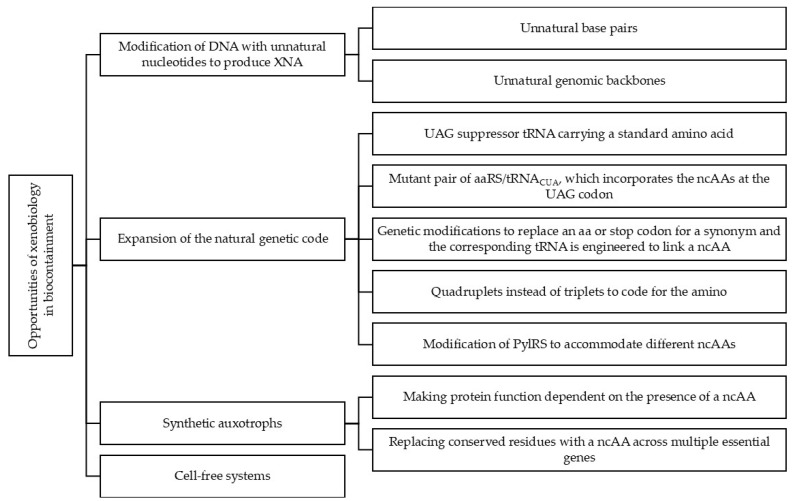
Main opportunities of xenobiology in biocontainment.

Xenobiology, with its approach of overcoming the current paradigms of the biochemistry of organisms, represents a very attractive option in the development of novel forms of biocontainment (Figure 1) since the synthetic elements in the organisms could not interact with the natural elements of the organisms in which they are inserted, nor with those of other organisms in the same environment, and are genuinely orthogonal, what has been called “semantic containment” [15].

In engineering, orthogonality means that modifying one component of a given system has no influence on other components of the system. This offers a clear benefit if the intention is to create an independent system that coexists with a natural one. The implications for biosafety of that system are very important since the orthogonal (unnatural) system could be controlled in different ways [14]. Importantly, according to the National Institutes of Health (NIH), a biocontainment system is regarded as safe below one escapee in a population of 10^8^ cells [16]. The two approaches followed in Xenobiology, i.e., the introduction of XNA in the genome or the alteration of the genetic code to incorporate non-canonical amino acids (ncAA), can be useful to develop biocontained synthetic organisms due to the different physicochemical character (DNA and RNA) or decoding logic (transcription and translation) in their genetic code as compared with natural organisms, which rules out the possibility of horizontal gene transfer (HGT) [17,18]. Some authors, when studying possible chemistries for hypothetical extraterrestrial life, which is closely related to the concept of xenobiology, have pointed out the importance of the hydrogen bond network for the generation of alternative biochemistries [19]. In fact, new methodologies are being tested to study the stability of this type of bonding in natural and unnatural base pairing [20]. Therefore, the possibilities regarding the design of alternative biochemistries are limited by the need to rely on hydrogen bonding [19].

As mentioned above, one possibility is the modification of DNA with unnatural nucleotides to produce XNA, either by means of unnatural base pairs or unnatural genomic backbones [14,21,22]. Replication and transcription of XNA was already achieved a decade ago [23,24]. Later, in 2017, it was reported that the transcription and translation of the first protein which incorporates non-canonical amino acids (ncAA) from a xenobiological genetic code was achieved, opening a door to accessing a wide range of forms and functions unavailable to natural organisms [25]. In 2019, DNA- and RNA-like systems containing eight different nucleotides were obtained, which were called “hachimoji”. Hachimoji systems contained the four natural nucleotides (A, C, G, T) and four artificial ones (Z, P, S, and B), forming four complementary pairs instead of two. Hachimoji DNA was then transcribed to give hachimoji RNA and, according to the authors, it meets the structural requirements needed to support Darwinism [26]. Another strategy is based on matching by hydrophobic forces. In this case the unnatural base pairs change the essence of the bonds between them, binding between themselves by means of hydrophobic forces and not by hydrogen bridges. The Hirao and Romesberg groups are prominent in this strategy. Hirao was able to select high affinity aptamers with unnatural bases with applications for the detection of diseases [27]. Romesberg was the first to insert an unnatural base pair of hydrophobic nature in a plasmid that replicates in Escherichia coli generating the first semi-synthetic organism (SSO) [28].

The introduction of new nucleotides in the DNA allows expanding the number of codons [29,30]. However, a more studied possibility is the expansion of the natural genetic code to include ncAAs, that is the expansion of the genetic code by means of the reassignment of codons, which allows the insertion of ncAAs into protein sequences [31]. “Ideally, estranging the genetic code from its current form via systematic introduction of ncAAs should enable the development of bio-containment mechanisms in synthetic cells potentially endowing them with a “genetic firewall” i.e., orthogonality which prevents genetic information transfer to natural systems” [12]. Kato defines three generations of methods, the first based on a UAG suppressor tRNA (tRNA_CUA_) carrying a standard amino acid, the second uses a mutant pair of orthogonal aminoacyl-tRNA synthetase (aaRS)/tRNA_CUA_, which specifically incorporates the ncAAs at the UAG codon, and the third improves on the previous one by including genetic modifications for switching uses. In these methods, transcription and protein synthesis can be induced with changes of temperature, the presence of some regulators, controlling the Aminoacyl-tRNA Synthetase, or making transient transfections of a tRNA_CUA_ expression construct [32]. The most common strategy is to select an amino acid or stop codon that is encoded by more than one triplet and to edit the genome to replace one of those triplets for a synonym. Once this is done, the corresponding tRNA of the vacated triplet can be engineered to link a ncAA [13]. A remarkable example of this approach was the recoding of 18,214 codons (representing two types of sense codons and one stop codon) in the genome of *E. coli* to create an organism with a 61-codon genome [33]. Additionally, it could also be possible for quadruplets instead of triplets to code for the amino acids, which would theoretically allow for 256 different assignments, opening the door to a significant increase in amino acids that could be incorporated to generate a multitude of novel proteins [34].

Also, a natural exception to the genetic code has been studied for its potential to be used to incorporate ncAAs. This exception is the amino acid pyrrolysine, which some methanogenic archaea and bacteria use in the biosynthesis of their proteins. This amino acid is encoded by the codon UAG (normally a stop codon) facilitated by pyrrolysyl-tRNA synthetase (PylRS) and the cognate UAG-recognizing tRNA^Pyl^ [35]. This system of tRNA and aminoacyl-tRNA synthetase (aaRS) can be used in different organisms as an orthogonal translation system (OTS). Remarkably, PylRS has an active site that can be easily modified by means of directed evolution to accommodate different ncAAs and it specifically recognizes the cognate tRNA^Pyl^ in an anticodon-blind way. These characteristics make PylRS a very interesting option for genetic code expansion and have already been used to incorporate more than 100 different ncAAs into proteins [36]. A technology that fuses solubility tags to the PylRS sequence improves enzyme solubility and boosts orthogonal translation efficiency, enhancing the production of site-specifically labelled proteins [37].

Another approach which can complement the former to improve biosafety is the generation of synthetic auxotrophs, whose growth depends on exogenously supplied artificial amino acids, which represents a form of containment regarding their inability to survive in the natural environment. To minimize the escape frequency (EF) of GROs, the organism must be highly dependent on the incorporation of the ncAA, what has been called “addiction to ncAAs” [38]. The creation of synthetic safeguard strains whose survival is dependent on ncAAs has been proposed as an effective method to introduce orthogonal barriers between GMOs and the environment and to reduce the risk of spreading them into the open environment, as well as a promising solution to minimize the risk of espionage activities in industrial applications [39,40]. Also, after the creation of the first fully orthogonal ribosome−mRNA system [41], an orthogonal ribosome (O-ribosome)-based firewall which includes activation and degradation systems has been proposed as a versatile, efficient, and flexible biocontainment system [42].

The addiction of protein function to a synthetic amino acid can be achieved by two main routes, namely, making protein function dependent on the presence of a ncAA or replacing conserved residues with a ncAA across multiple essential genes [38]. Examples of the first approach include: the construction of an *E. coli* strain that cannot survive in the absence of the ncAA 3-iodo-L-tyrosine, as it was necessary to produce the antidote protein against the highly toxic enzyme colicin E3 (a plasmid with the pair toxin-antidote was also introduced) [43]; the computational redesigning of hydrophobic interactions in protein cores to exclusively accommodate 4,4′-biphenylalanine (bipA), which resulted in low EF (≈10^−8^) of two organisms recoded for the genes of adenylate kinase and tyrosyl-tRNA synthetase, respectively, and no escape variants detected (EF < 10^−12^) of an organism that harbored the mutations for both genes [44]; directed evolution to make the hydrophobic packing of protein cores depend on a ncAA, such as the reengineering of TEM-1 β-lactamase to be dependent on a 3-nitrotyrosine (3nY) or 3-iodotyrosine (3iY) in *E. coli*, without detecting any escape variants (EF < 10^−11^) [45]; replacement of a natural active site residue with a non-standard one, such as the introduction of Nϵ-acetyllysine (AcK) into the essential branched chain aminotransferase (BCAT) of *E. coli*, where a conditional kill switch was required to obtain an EF < 10^−11^, since escape mutants appeared which had a point mutation in the anticodon of an *E. coli* lysine tRNA (3′-UUU-5′ to 3′-AUU-5′), resulting in an anticodon that could suppress UAG stop codons through G/U wobble pair formation [46]; and the directed evolution of an *E. coli* sliding clamp variant with an orthogonal protein-protein interface which contains p-benzoylphenyl alanine (pBzF), achieving a low EF < 10^−10^ [47]. As an example of the second approach, Rovner, et al. [10] constructed a series of GROs whose growth was restricted by the expression of several essential genes dependent on exogenously supplied synthetic phenylalanine-derived amino acids. They used a GRO that lacks all TAG codons and release factor 1 and introduced a *Methanocaldococcus jannaschii* tRNA: aminoacyl-tRNA synthetase pair into its chromosome, endowing this organism with the orthogonal translational components to convert TAG into a sense codon for sAAs. In-frame TAG codons were introduced into 22 essential genes. With this approach they achieved the generation of synthetic auxotrophs which exhibited robust growth and undetectable escape frequencies and were not rescued by cross-feeding in environmental growth assays, since many of the chosen essential genes had functions that cannot be complemented by cross-feeding of metabolites (e.g., replication, translation).

Despite the important progress in bacteria, until very recently there was no example of development of ncAA-dependent eukaryotic microorganisms. Chang et al. [40] have developed a robust yeast biocontainment system for the first time using the orthogonal aaRS/tRNA pair (LeuOmeRS/tRNACUA) to site-specifically incorporate O-methyltyrosine (OMeY) into essential proteins in response to the UAG stop codon. Interestingly, this study suggests that disruption of nonsense-mediated mRNA decay (NMD), a eukaryotic surveillance system, is a major escape route of OMeY-dependent *S. cerevisiae* strains. Nevertheless, the authors acknowledge a key limitation of their biocontainment strategy due to the elevated cost of the unAA supplementation for large-scale fermentation suggesting that although this additional cost could be acceptable for biosynthesis of high value-added products, it will be desirable to develop strains able to grow at a low concentration of micromolar concentrations of unAA. The horizon of this research is the development of a robust yeast biocontainment system dependent on unAA in the upcoming final Sc2.0 strain, the product of the Synthetic Yeast Genome Project (Sc2.0), a global consortium working to develop the first synthetic eukaryote genome from scratch [48].

Synthetic auxotrophy can also be used to control the expression of certain genes, in an approach that, more than the biocontainment of synthetic organisms, seeks the biocontainment of certain synthetic genetic circuits, so that the translation of a certain protein is conditioned to the availability of a ncAA [49]. Additionally, ncAA dependency can also be used to control viral replication, which in turn can be used to generate live-attenuated vaccines (LAVs). The strategy involves the introduction of multiple stop codons in the genome of viruses which will be translated into a ncAA. Therefore, in the absence of the ncAA, the virus remains alive but replication incompetent, which means that it will generate an immune response but will not proliferate [50].

A recent publication incorporates CRISPR to the array of synthetic auxotrophy technologies being used in the design of biocontainment strategies [51]. This Cas9-assisted auxotrophic biocontainment system combines thymidine auxotrophy, a riboregulator to control gene expression, and a CRISPR device that prevents the bacteria from breaking the containment by horizontal gene transfer. Approaches like this one could pave the way for a safe use of genetically engineered microorganisms in biomedicine.

An alternative approach was used by Fujino et al. to specifically prevent the escape of genes (instead of preventing the leakage of GMOs.) They developed amino acid (AA)-swapped genetic codes orthogonal to the standard genetic code, namely SL (Ser and Leu were swapped) and SLA genetic codes (Ser, Leu, and Ala were swapped). The mRNAs encoded by the AA-swapped genetic codes only were properly translated into functional proteins in translation systems featuring the corresponding genetic codes [52].

Finally, cell-free systems (CFS) based on Xeno-DNA may represent the ultimate biosafety system [53]. CFS, also known as cell-free protein synthesis (CFPS), has been already used in multiple experiments, including the incorporation of orthogonal genetic codes [54,55] and ncAAs via codon reassignment [56]. Reassigned codon usage forms a strong barrier against genetic contamination [39]. In general, these approaches exhibited a lower biosafety risk than with living systems, efficiently restricting the spread of the uptake or dispersal of transgenic materials and showing promise as a biocontainment strategy [57]. More recently, the cell-free system has evolved into portable and convenient biomanufacturing platforms, solving challenges for the delivery of therapeutics where delicate storage needs are required [58]. This portable, in situ characteristic of cell-free platforms are of special interest for biosafety issues in synthetic biology. Nevertheless, we should consider that some CFS might be deliberately deployed to the environment for biosensing and remediation, requiring improvements in reagent shelf-life and optimizing encapsulation to facilitate practical use and increase its biosafety [59]. Interestingly, some authors have shown that even dried CFPS reactions are robust during the casting processes for polymer materials, unlocking the possibility to deliver DNA-programmable biofunctions in synthetic materials [60,61].

## 3. Challenges of Xenobiology in Biocontainment

**Figure 2:**
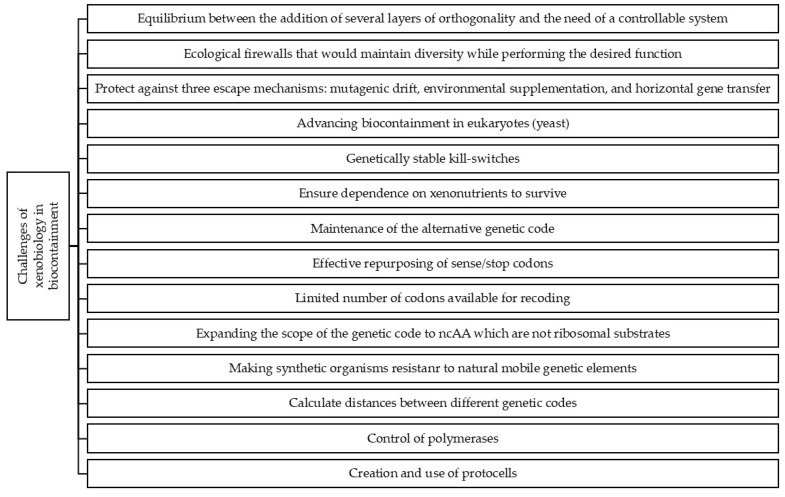
Main challenges of xenobiology in biocontainment.

Although physical containment (bioreactors or microencapsulation) has been proposed as a first layer of containment, additional biological mechanisms are recognized to be essential for an efficient biocontainment [62]. In order to ensure biosafety, Schmidt [14] proposes some biological and technical specifications such as the importance of maintaining auxotrophy, the inability of natural polymerases to replicate and transcribe XNA to DNA or RNA and vice versa, the “invisibility” of XNA genes for natural transcription factors, the need to avoid a merging between natural and artificial cells, or the convenience for the artificial molecules to be recyclable once they are not needed. He also suggests the convenience of adding additional layers of orthogonality to improve biosafety (Figure 2).

Schmidt [14] proposes, for an effective genetic firewall, that XNA nucleotides should be at least two steps away from any natural molecule, that replication of the orthogonal system should be invisible (different machineries required) for the natural one and vice versa, and finally a desirable increase in orthogonality with different unnatural architectures. Importantly, genetic firewalls are not necessarily biological firewalls since interaction between DNA and XNA organisms on an ecological level would be possible. Even though the genetic firewalls are effective, XNA organisms could be allowed to form their own, although controlled, ecosystems and even share them with the natural organisms but with a strict condition of auxotrophy. However, if we increase the complexity of a safety system or genetic firewall, we are probably increasing the chance of failure. So, it is important to consider an equilibrated approach between the addition of several layers of orthogonality and the need of a controllable system [62].

Some authors propose to consider the different types of ecological interactions to design ecological firewalls that would maintain diversity while performing the desired function [63]. Its aim is to stabilize a community of synthetic microorganisms able to degrade an undesired anthropogenic substrate. We suggest that a combination of this ecological approach with advanced xenobiological firewalls like the ones proposed by Schimdt [14], could generate a more ecological strategy for an effective biocontainment.

Effective biocontainment strategies must protect against three possible escape mechanisms: mutagenic drift, environmental supplementation, and horizontal gene transfer (HGT). Distribution of multiple non-canonical amino acid-dependent enzymes throughout a genome would be an efficient strategy to prevent escape by HGT since conjugal escape would require the replacement of large portions of the recipient genome, making the escape very unlikely [39,44].

Although these technologies have been thoroughly studied in *E. coli*, in yeasts, which are essential in industrial biotechnology, the complexity of the eukaryotic genome, the intracellular compartmentalization and biochemical specialization of organelles, and its richness in mechanisms that increases its genetic diversity could lead to the inactivation of biocontainment mechanisms, making necessary the development of new biocontainment approaches in these organisms [64]. The development of synthetic biology programs like “Sc 2.0” [65,66] which is designed to reassign the TAG stop codon to ncAA could be used to test the ncAA-dependent biocontainment strategy that ensures effective firewalls in these organisms [40].

Since genetic instability of synthetic genetic devices such as kill-switches for conditional host killing is a key obstacle for practical use, some authors have recently proposed an improved version of a genetically stable kill-switch based on what they call a “demon and angel” expression construct of a toxic essential gene. This essential gene is expressed at a low level to maintain host survival in the OFF state and kills the host by the overexpression in the ON state. Interestingly, they found that toxic overexpression of essential genes has also been found in other organisms, suggesting that this kill switch could be scalable to various organisms [67].

Regarding xenobiology approaches that intend to transform nucleic acids, there is no experimental evidence that organisms engineered in this sense would absolutely depend on xenonutrients to survive, and then vertical gene transfer (VGT) could occur in an open environment [17]. Additionally, the design of reverse transcriptases that convert either between different XNAs, between XNA and DNA, or between XNA and RNA, could at one point overcome the orthogonality [68].

Also, regarding the expansion of the genetic code, it has not yet been possible to generate an organism that would use and maintain an alternative genetic code permanently [69]. Although cell-free experiments have demonstrated the feasibility of substituting natural amino acids with unnatural alternatives in the protein structure, achieving this in living cells remains elusive due to several biological restrictions, such as the physicochemical properties of ncAAs and their role in protein folding, metabolic integration, and participation in regulatory networks [12]. To circumvent this challenge, consideration is being given to the chemical logic behind the amino acid repertoire establishment in the RNA world [70,71], which can shed light into the artificial expansion of the genetic code in a good direction [12]. Kubyshkin and Budisa [12] have chosen the theory of Hartman and Smith [70], among other possible models to explain the evolution of the genetic code, because it focuses on the chemical logic behind the actual amino acid pool available in organisms, thus providing a useful framework to predict “entry points” for ncAAs in xenobiology research. These authors consider that the “most feasible approach is suitably designed evolution experiments by using proper metabolic prototypes that enable cells to adapt and overcome harmful effects caused by the incorporation of the non-canonical amino acid(s)” [12].

As explained before, there are two mechanisms to incorporate ncAAs in organisms: repurposing stop codons or repurposing a given sense codon. The problem with repurposing a stop codon is that while the ncAA incorporation is achieved in a protein of interest, the same stop codon appears in different transcripts, whose unnatural decoding may have deleterious consequences. Additionally, protein production via stop codon suppression is hindered by release factors, as they compete with the suppressor tRNA for binding to the same stop codon. This issue can be partially alleviated by using strains with release factor deletions. On the other hand, if sense codons are repurposed instead of stop codons, these problems are overcome. However, the orthogonal aaRS should not recognize the tRNA anticodon and the orthogonal tRNA should not be recognized by a natural aaRS in the cell [36]. As an example, serine and leucine anticodons were introduced into tRNA^Pyl^ in *E. coli*. As neither the *E. coli* LeuRS or SerRS recognize the tRNA anticodon, they did not cross-react with tRNA^Pyl^_CAG_ or tRNA^Pyl^_ACU_, resulting in orthogonal tRNA^Pyl^ variants [72]. Also degenerate codons can be targeted, an approach that requires elimination of the host’s corresponding tRNA. In all cases, an additional constraint is the need for an orthogonal tRNA, for which some mutagenesis can be required.

Also, the simultaneous incorporation of several ncAAs is not efficient, since adding codons to the genetic code is not straightforward and the number of codons readily available for recoding is limited. In this regard, orthogonal ribosomes (ORs), which have mutations in the 16S ribosomal RNA, could constitute a useful tool, since parallel genetic codes could be introduced and read only by these ORs, while ORs would not recognize native mRNAs, thus reinforcing synthetic biocontainment [38].

A limitation of the traditional approaches to incorporate ncAA into proteins is that these amino acids must be ribosomal substrates (α-L-amino acids and closely related hydroxy acids). In order to circumvent this limitation, researchers have developed tRNA display, a method that allows for direct selection of orthogonal synthetases that selectively acylate their cognate orthogonal tRNAs with other non-canonical monomers (ncMs) in Escherichia coli, including several β-amino acids, α,α-disubstituted-amino acids, and β-hydroxy acids, expanding the chemical scope of the genetic code to new classes of monomers [73].

An additional biosafety consideration is that, in addition to the need to prevent escape of genetic material from modified organisms into natural organisms, it is also necessary for synthetic organisms to be resistant to natural mobile genetic elements. Therefore, refactoring the genetic code to block the invasion of mobile genetic elements should complement strategies to control the survival and growth of synthetic organisms, which is especially important when considering applications of engineered organisms outside the laboratory. According to recent research, the GRO with altered genetic code is immune to viral infections and genetic elements that are transferred [74,75].

An unresolved issue is what differences must the two genetic codes have to be truly orthogonal among each other. It is assumed that the greater the difference between genomes, the less likely they are to interact (“the farther the safer” premise). However, it is unclear how to measure that distance and how far would be safe enough [13]. “Despite the claim that synthetic biology is the true application of engineering principles to biotechnology, right now there are surprisingly few metrics (and standards) available” [68]. A metric to calculate distances between genetic codes should fulfill two criteria: to be universal (applied to all organisms) and to be biologically meaningful (capable of reflecting the biological implications of the changes made) [13]. Schmidt and Kubyshkin [68] developed a metric (Δcode) to calculate distances between different genetic codes. In their metric, polarity indices (clog D 7) are assigned to amino acids to calculate the distances between pairs of genetic codes. This tool could be useful in the generation of “semantically alienated organisms” as well as in “testing the strength of genetic firewalls” [62]. However, this metric is only applicable for codes who share the same base alphabet (e.g., A, U, G, C) and codon length (e.g., 3). Other xenobiology approaches such as modification of the codon length, the nucleic acid structure, or the base pairs, lack this type of metric. Future research is needed to unravel what distance would be necessary to achieve 100% security. This research involves the generation of different GRO and their evaluation in horizontal gene transfer tests (for example selection under antibiotic resistance) [13].

Directed evolution is a promising approach for the development of XNA replicating systems, where new polymerases are needed for the processing of the information [14]. A promising method of directed evolution is the so called compartmentalized self-replication (CSR). CSR is based on a simple feedback loop within a simple vesicle, in which a polymerase replicates only its own encoding gene [76]. Nevertheless, an important issue to be considered in the development of safe orthogonal systems is the fact that small changes in polymerases can lead them to read novel genetic codes [77]. Another approach to increase the safety of orthogonal systems is the creation and use of protocells that could be designed with a completely new chemical architecture different from the central dogma [78].

Although a wide array of strategies has been tested for orthogonal genetic systems in synthetic biology applications [79], the construction of a fully host-orthogonal genetic system, with all the parts operating in an orchestrated, integrated, and controlled manner, still poses an extraordinary challenge for the researchers.

## 4. Conclusions and Future Directions

Xenobiology offers exciting opportunities for the development of synthetic organisms with innovative biocontainment mechanisms. Current strategies include the introduction of XNA into the genome and, especially, the alteration of the genetic code to incorporate ncAAs, allowing the establishment of unnatural auxotrophies. These strategies could offer safe and controllable biological systems. However, significant challenges are faced, such as the need for multiple layers of orthogonality to improve biosafety and the complexity of ensuring ncAA dependence. In the coming years, research will be necessary to balance safety with functionality in synthetic cells, gain evidence regarding the safety of these systems, which is still limited, improve the use and dependence of ncAAs, and develop cell-free systems for those applications in which its use is feasible. Although more research and development are required, the potential for creating safe and functionally useful synthetic organisms is promising.
